# Quantitative real-time PCR assay for the rapid identification of the intrinsically multidrug-resistant bacterial pathogen *Stenotrophomonas maltophilia*

**DOI:** 10.1099/mgen.0.000307

**Published:** 2019-10-16

**Authors:** Tamieka A. Fraser, Mikaela G. Bell, Patrick N. A. Harris, Scott C. Bell, Haakon Bergh, Thuy-Khanh Nguyen, Timothy J. Kidd, Graeme R. Nimmo, Derek S. Sarovich, Erin P. Price

**Affiliations:** ^1^​ GeneCology Research Centre, University of the Sunshine Coast, Sippy Downs, Queensland, Australia; ^2^​ Sunshine Coast Health Institute, Birtinya, Queensland, Australia; ^3^​ Microbiology Department, Central Laboratory, Pathology Queensland, Royal Brisbane and Women’s Hospital, Herston, Queensland, Australia; ^4^​ University of Queensland Centre for Clinical Research, Royal Brisbane and Women’s Hospital, Herston, Queensland, Australia; ^5^​ QIMR Berghofer Medical Research Institute, Herston, Queensland, Australia; ^6^​ Adult Cystic Fibrosis Centre, Prince Charles Hospital, Chermside, Queensland, Australia; ^7^​ School of Chemistry and Molecular Biosciences, Faculty of Science, University of Queensland, St Lucia, Queensland, Australia

**Keywords:** *Stenotrophomonas maltophilia*, diagnostics, comparative genomics

## Abstract

*
Stenotrophomonas maltophilia* is emerging as an important cause of disease in nosocomial and community-acquired settings, including bloodstream, wound and catheter-associated infections. Cystic fibrosis (CF) airways also provide optimal growth conditions for various opportunistic pathogens with high antibiotic tolerance, including *S. maltophilia *. Currently, there is no rapid, cost-effective and accurate molecular method for detecting this potentially life-threatening pathogen, particularly in polymicrobial specimens, suggesting that its true prevalence is underestimated. Here, we used large-scale comparative genomics to identify a specific genetic target for *S. maltophilia *, with subsequent development and validation of a real-time PCR assay for its detection. Analysis of 167 *Stenotrophomonas* spp. genomes identified a conserved 4 kb region in *S. maltophilia *, which was targeted for Black Hole Quencher assay design. Our assay yielded the positive detection of 89 of 89 (100%) clinical *S. maltophilia* strains, and no amplification of 23 non-* S. maltophilia* clinical isolates. *S. maltophilia* was detected in 10 of 16 CF sputa, demonstrating the assay's utility for direct detection in respiratory specimens. The assay demonstrated good sensitivity, with limits of detection and quantitation on pure culture of ~10 and ~100 genome equivalents, respectively. Our assay provides a highly specific, sensitive and cost-effective method for the accurate identification of *S. maltophilia *, and will improve the diagnosis and treatment of this under-recognized pathogen by enabling its accurate and rapid detection from polymicrobial clinical and environmental samples.

## Data Summary

All 167 genomes used for reconstructing *Stenotrophomonas* spp. phylogeny are described in (available with the online version of this article), including accession numbers obtained from the National Center for Biotechnology Information Sequence Read Archive (SRA) database (https://www.ncbi.nlm.nih.gov/sra).

**Impact Statement**

An over-reliance on antibiotics in recent decades has led to the unintended rise of antimicrobial-resistant opportunistic bacterial pathogens that are difficult to treat. Incorrect or under-diagnosis of pathogens, especially naturally antimicrobial-resistant species, has dire consequences for patient health and effective antibiotic stewardship measures. One example is *S. maltophilia *, a bacterium with naturally high antibiotic tolerance that is increasingly being recognized as a life-threatening pathogen, particularly in nosocomial settings. Current methods for identifying *S. maltophilia* rely on selective culture or mass spectrometry﻿, neither of which can reliably detect *S. maltophilia* in polymicrobial or low-abundance specimens. Here, we use a large-scale comparative genomics approach to reconstruct the phylogeny of *Stenotrophomonas* spp. from 167 publicly available genomes, with subsequent identification of a conserved 4 kb region of *S. maltophilia *, which was targeted for real-time PCR assay development. We demonstrate that our new assay is sensitive and specific, and provides accurate, cost-effective and rapid detection of *S. maltophilia* from cultures and polymicrobial specimens. The real-time PCR format provides an accessible way to accurately assess the true prevalence of *S. maltophilia *, which is essential for guiding antibiotic treatment options and improving patient outcomes. Finally, our study highlights the utility of comparative genomics for robust taxonomic reassignment of speciation errors that are, unfortunately, common in public databases.

## Introduction

*Stenotrophomonas maltophilia* is a Gram-negative, intrinsically multidrug-resistant bacterium that is ubiquitous in aqueous environments, such as soils, plant roots, and water treatment and distribution systems [1]. Whilst conventionally overlooked as a laboratory contaminant, or as a common commensal in hospitalized patients, *S. maltophilia* is increasingly being recognized as an important nosocomial pathogen in its own right, due to its ability to cause life-threatening disease in immunocompromised individuals [2]. This opportunistic pathogen has been isolated from a variety of hospital settings, including taps, sinks, central venous catheters, ice machines, and water fountains, reinforcing its nosocomial importance [1, 3, 4]. *S. maltophilia* most commonly infects people with meningitis, cancer, chronic obstructive pulmonary disease or cystic fibrosis (CF), with pneumonia, bacteraemia, and wound and urinary infections being the most frequent clinical manifestations [5, 6]. Risk factors for *S. maltophilia* infection include prolonged hospitalization, neutropenia, catheterization and previous use of broad-spectrum antibiotics [7]. The recommended antibiotic treatment for *S. maltophilia* infections is co-trimoxazole; however, resistance towards this antibiotic combination has been documented [2, 8, 9]. Indeed, treatment options are limited for *S. maltophilia *, with this pathogen also exhibiting resistance towards several antibiotic classes, including fluoroquinolones, macrolides, β-lactams, aminoglycosides, carbapenems, tetracyclines, polymyxins, chloramphenicol and cephalosporins [1, 2]. With a mortality rate approaching 70 %, the importance of timely identification and effective treatment of *S. maltophilia* infections is paramount [10].

*S. maltophilia* is a common pathogen in CF airways due to its ability to evade many antipseudomonal antibiotics, with chronic *S. maltophilia* infection associated with an increased risk of respiratory disease and mortality [11, 12]. CF is an autosomal recessive genetic disorder effecting multiple organs; however, its pathogenesis is most prominent in airways, with ~90 % of CF deaths associated with respiratory failure [13]. The excessive production of mucus in CF airways provides optimal growth conditions for opportunistic pathogens, which drives most CF morbidity and mortality. Molecular methods have confirmed that CF lower airways harbour diverse microbial communities, with *Pseudomonas aeruginosa* and *Burkholderia cepacia* complex species of greatest concern due to frequent rapid respiratory decline in people infected with these pathogens [14, 15]. However, other co-infecting opportunistic pathogens, such as *Achromobacter* spp., *S. maltophilia *, *Staphylococcus aureus *, *Haemophilus influenzae* and certain fungal species (e.g. *Aspergillus*), are also prominent in CF airways, and are known to contribute to pathogenesis [14]. Indeed, recent studies have shown a mutualistic relationship between *S. maltophilia* and *P. aeruginosa* in CF airways, with compounds produced by *S. maltophilia* under exposure to certain antibiotics, such as imipenem, supporting the survival of otherwise antibiotic-susceptible *P. aeruginosa* strains [16]. Furthermore, these *S. maltophilia* compounds can enhance *P. aeruginosa* stress tolerance, increasing polymyxin tolerance [17]. These studies highlight the importance of *S. maltophilia* in CF airway pathogenesis, particularly during antibiotic treatment, and emphasize the need for correct species identification in polymicrobial infections.

Although there are a variety of diagnostic methods available for *S. maltophilia* detection, such as PCR amplicon sequencing, VITEK MS identification or key morphological characteristics on growth media, these methods suffer from issues such as limited access to equipment with a large capital expenditure [e.g. ~US $200 000 (£162 586, £1=$1.23) for VITEK MS instrumentation], high per-assay cost, the need for highly trained personnel, laboriousness, slow turnaround time, the requirement for purified colonies, and misidentification issues [16, 18, 19]. For example, *S. maltophilia* and *P. aeruginosa* exhibit colony colour differences when grown on bromothymol blue-containing media, which reflects their different metabolic processes [16]. However, the use of media containing bromothymol blue is not routine and, thus, the retrieval of *S. maltophilia* from polymicrobial specimens requires clinical expertise in identifying appropriate culture media for differentiation of this bacterium from other pathogens. As a non-exhaustive list, *S. maltophilia* has been misidentified as several other organisms, including *Bordetella bronchiseptica *, *Alcaligenes faecalis *, *Burkholderia cepacia* and numerous *Pseudomonas* species, which are common in clinical settings, including CF sputa [1, 20]. Diagnostic inconsistencies in *S. maltophilia* detection from clinical specimens can lead to inappropriate or even detrimental treatment [16], particularly for those patients requiring urgent care. Therefore, there is a need to accurately identify this emerging pathogen to improve antibiotic-treatment regimens, stewardship and patient outcomes. Here, we report the development and validation of a black hole quencher (BHQ) probe-based real-time PCR assay for the specific detection of *S. maltophilia *. Our results indicate that our real-time PCR assay is more sensitive than routine culture for detecting *S. maltophilia *, particularly in polymicrobial respiratory specimens.

## Methods

### Genomes of *Stenotrophomonas* spp. and closely related species

All non-redundant *Stenotrophomonas* spp. genomes available in the National Center for Biotechnology Information (NCBI) that were generated using paired-end Illumina sequencing were downloaded from the Sequence Read Archive database (SRA; https://www.ncbi.nlm.nih.gov/sra) as of December 2018. Additional non-redundant *Stenotrophomonas* spp. genomes were downloaded from the NCBI GenBank database. In total, 167 publicly available genomes were accessible for this study (Table S1), represented by *S. maltophilia* (including all '* Stenotrophomonas pavanii *'; *n*=132), *Stenotrophomonas acidaminiphila* (*n*=4), *Stenotrophomonas bentonitica* (*n*=3), *Stenotrophomonas chelatiphaga* (*n*=1), *Stenotrophomonas daejeonensis* (*n*=1), *Stenotrophomonas ginsengisoli* (*n*=1), *Stenotrophomonas humi* (*n*=1), *Stenotrophomonas indicatrix* (*n*=6), *Stenotrophomonas koreensis* (*n*=1), *Stenotrophomonas lactitubi* (*n*=1), *Stenotrophomonas nitritireducens* (*n*=2), '* Stenotrophomonas panacihumi* *'* (*n*=1), *Stenotrophomonas pictorum* (*n*=1), *Stenotrophomonas rhizophila* (*n*=2), *Stenotrophomonas terrae* (*n*=1) and unassigned *Stenotrophomonas* spp. (*n*=9). Three strains listed on the NCBI database as *Pseudomonas geniculata* (95, AM526 and N1) were included in the phylogenomic analysis to confirm that they were in fact *S. maltophilia *. Sequence data from assembled genomes were converted to simulated 100 bp paired-end Illumina reads at 85× coverage using art version MountRainier [21]. SRA data were quality-filtered using Trimmomatic v0.33 [22] using previously described filtering parameters [23] prior to analysis.

### Bioinformatic analysis to identify a conserved *S. maltophilia* genetic locus not found in other *Stenotrophomonas* spp.

Default settings of the haploid comparative genomics pipeline SPANDx v3.2.1 [24] were used to phylogenetically delineate *S. maltophilia* from non-* S. maltophilia* species and, subsequently, to identify *S. maltophilia *-conserved loci. Illumina reads for the publicly available strains (Table S1) were mapped to the closed 4.85 Mbp *S. maltophilia* K279a genome (GenBank accession no. NC_010943.1) [25]. Phylogenomic reconstruction of the 167 taxa was performed using 31 246 core, biallelic SNPs using the maximum parsimony function of paup* v4.0a.164 [26], rooted with *S. daejeonensis* JCM 16244 (GenBank accession no. LDJP01000001.1), and with bootstrapping carried out using 1000 replicates. To identify genetic loci present in all *S. maltophilia* but absent in all other organisms, including other *Stenotrophomonas* spp., the BEDcov output [27] from SPANDx was examined. Candidate regions were assessed further for specificity and PCR assay design.

To identify the origin of the formate dehydrogenase loci in *S. indicatrix* RS1 and RS7 strains, alignment of the RS7 (GenBank accession no. RKSR01000015.1) contig encoding this genetic region (~117 kb) was compared with the K279a genome using progressiveMauve v20150226 build 10 [28].

### *S. maltophilia* probe-based real-time PCR assay design

Upon identification of a putative *S. maltophilia *-specific genetic target, sequence alignments were used to locate conserved regions. Although some variation was allowed within the amplicon, primer- and probe-binding regions required 100 % sequence identity in all *S. maltophilia* strains to avoid false negatives. Oligo self-dimers and heterodimers were assessed *in silico* using NetPrimer (http://www.premierbiosoft.com/netprimer/) and Beacon Designer (http://www.premierbiosoft.com/qOligo/Oligo.jsp/), with configurations resulting in ΔG values of <−8.0 (NetPrimer) and <−4.0 (Beacon Designer) excluded. The following sequences and probe label were chosen for the assay: Smalto-For 5′-AAGGACAAGGCGATGACCATC-3′, Smalto-Rev 5′-CCCCACCACGAYTTCATCA-3′ and Smalto-Probe 5′-FAM-CAGAACGACATCTGGTTGGCG-BHQ1-3′, resulting in an amplicon length of 344 bp. NCBI microbial nucleotide Discontiguous MegaBLAST (http://blast.ncbi.nlm.nih.gov/) analysis of this amplicon was used to determine assay specificity for only *S. maltophilia *. No mismatches in the probe-binding site were tolerated in any *S. maltophilia* strain, whereas ≥2 mismatches in the probe-binding site were considered sufficient for conferring *S. maltophilia* specificity.

### Microbiological cultures and CF sputum DNA extractions

A total of 89 *S *. *maltophilia* isolates were obtained for PCR testing: *S. maltophilia* control strain LMG 957, 16 sputum-derived isolates cultured from individuals with CF identified either by amplified rRNA gene restriction analysis (*n*=8 [29]) or *in silico* multilocus sequence typing (*n*=8 [30]), and 72 isolates from various clinical presentations that had previously been identified as *S. maltophilia* by the Pathology Queensland Central Laboratory, Brisbane, Australia, according to the VITEK2 GNI card (98 % of isolates) or VITEK MS (2 % of isolates). Strains were grown on Luria–Bertani (LB) agar for 24 h at 37 °C. DNA was extracted from a small (toothpick head) swatch of the primary culture by heat soaking in 80 µl of a 5 % Chelex solution at 95 °C for 10 min, with the resultant DNA diluted 1 : 10 with molecular grade H_2_O prior to real-time PCR testing.

Sixteen sputa collected from nine Australian adult CF patients underwent DNA extraction using the Zymo Quick-DNA miniprep plus kit (ZYMO Research) protocol, with the addition of an enzymatic lysis solution (20 mg lysozyme ml^−1^, 22 U lysostaphin ml^−1^ and 250 U mutanolysin ml^−1^) at 37 °C for 1–3 h prior to the addition of Proteinase K. Three patients had sputa collected over three time points (range: 13–46 days), and one patient had two sputa collected 320 days apart. ‘Day 1’ samples represent sputa collected on the day of intravenous antibiotic commencement.

### *S. maltophilia* real-time PCR assay validation and specificity testing

Each PCR consisted of 1× SsoAdvanced universal probes supermix (Bio-Rad), optimized primer and probe concentrations of 0.30 and 0.35 µM, respectively (Macrogen), 1 µl DNA template, and RNase/DNase-free PCR grade water (Thermo Fisher Scientific), to a final per-reaction volume of 5 µl. Optimized thermocycling parameters included an initial hot start activation at 95 °C for 2 min, followed by 45 cycles of 95 °C for 3 s and 60 °C for 10 s, using the CFX96 Touch real-time PCR detection system (Bio-Rad). Results were analysed using CFX Maestro v4.1.2433.1219 software.

The 89 *S *. *maltophilia* isolates and 16 CF sputa were tested using the optimized PCR conditions to ensure accurate detection of *S. maltophilia* DNA in known positive isolates, and in sputum samples from which *S. maltophilia* was variably detected using selective culture (horse blood agar, McConkey agar, *Burkholderia cepacia* agar base and Bacitracin agar base). Relative *S. maltophilia* abundance in sputa was determined by performing a 16S rRNA gene real-time PCR as described elsewhere [31] and calculating the cycles-to-threshold difference (∆*C*_t_) between 16S rRNA gene and the *S. maltophilia* assays. Further specificity testing was performed on 28 non-* S. maltophilia* DNA: *Burkholderia thailandensis* (*n*=1), *Burkholderia territorii* (*n*=4), *Burkholderia cenocepacia* (*n*=1), *Enterobacter aerogenes* (*n*=1), *Enterobacter cloacae* (*n*=4), *Klebsiella oxytoca* (*n*=1), *Klebsiella pneumoniae* (*n*=2), *P. aeruginosa* (*n*=11), *Staphylococcus aureus* (*n*=1) and *Staphylococcus epidermidis* (*n*=2).

Limit of detection (LoD) and limit of quantification (LoQ) were determined for our newly designed *S. maltophilia* real-time PCR assay using *S. maltophilia* DNA serial dilutions of 50 ng µl^−1^ to 0.05 fg µl^−1^ across eight replicates per dilution. Twenty-four no-template controls were also included. The upper and lower LoD/LoQ limits were determined as described elsewhere [32].

## Results

### Comparative genomics of *Stenotrophomonas* spp. identifies a *S. maltophilia *-specific gene target

**Fig. 1.**
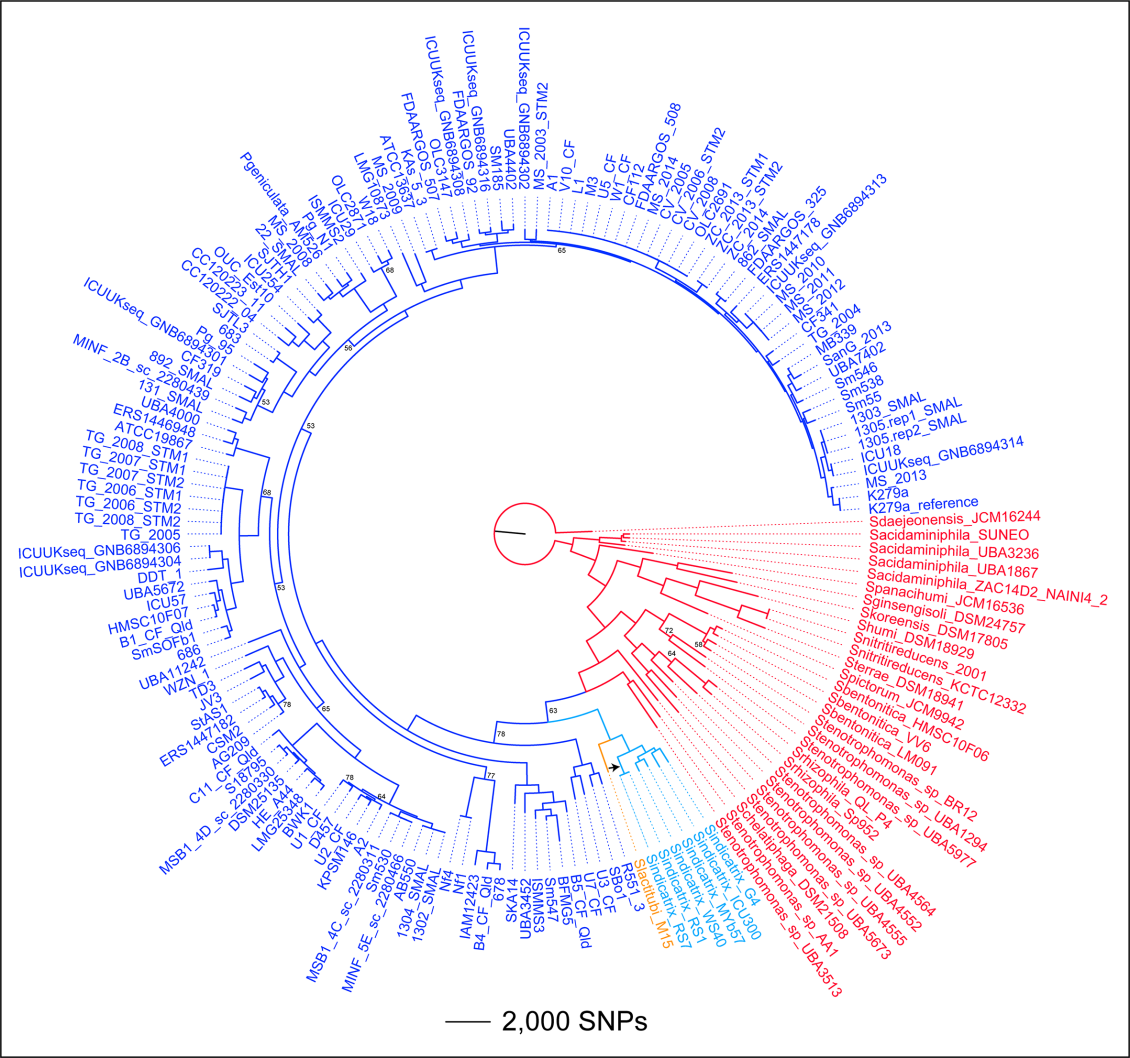
Phylogenomic analysis of *Stenotrophomonas* spp. Dark blue, *S. maltophilia* (target species); red, distantly related *Stenotrophomonas* spp.; light blue, *S. indicatrix *; orange, *S. lactitubi *. The tree was rooted with *S. daejeonensis* JCM16244. Consistency index=0.20. Branches with bootstrap values with <80 % support are labelled. The black arrow indicates the phylogenetic position of a probable homologous recombination event from an *S. maltophilia* strain to a recent common ancestor of the RS1–RS7 clade. This recombination event involved genes encoding formate dehydrogenase.

Phylogenomic reconstruction of 167 *Stenotrophomonas* spp. genomes using 31 306 core-genome, biallelic, orthologous SNPs demonstrated a close relationship between *S. maltophilia* and two recently described *Stenotrophomonas* species, *S. indicatrix* and *S. lactitubi* [33], and a clear distinction of these taxa from other *Stenotrophomonas* spp. (Fig. 1). This phylogenomic analysis was used to delineate *S. maltophilia* from other species, and to modify incorrect species designations for 43 taxa, including reclassification of all 4 '* S. pavanii *﻿', 3 *P. geniculata* and 17 *Stenotrophomonas* sp. as *S. maltophilia *, and 9 *S. maltophilia* as *Stenotrophomonas* spp. (Table S1).

**Fig. 2.**
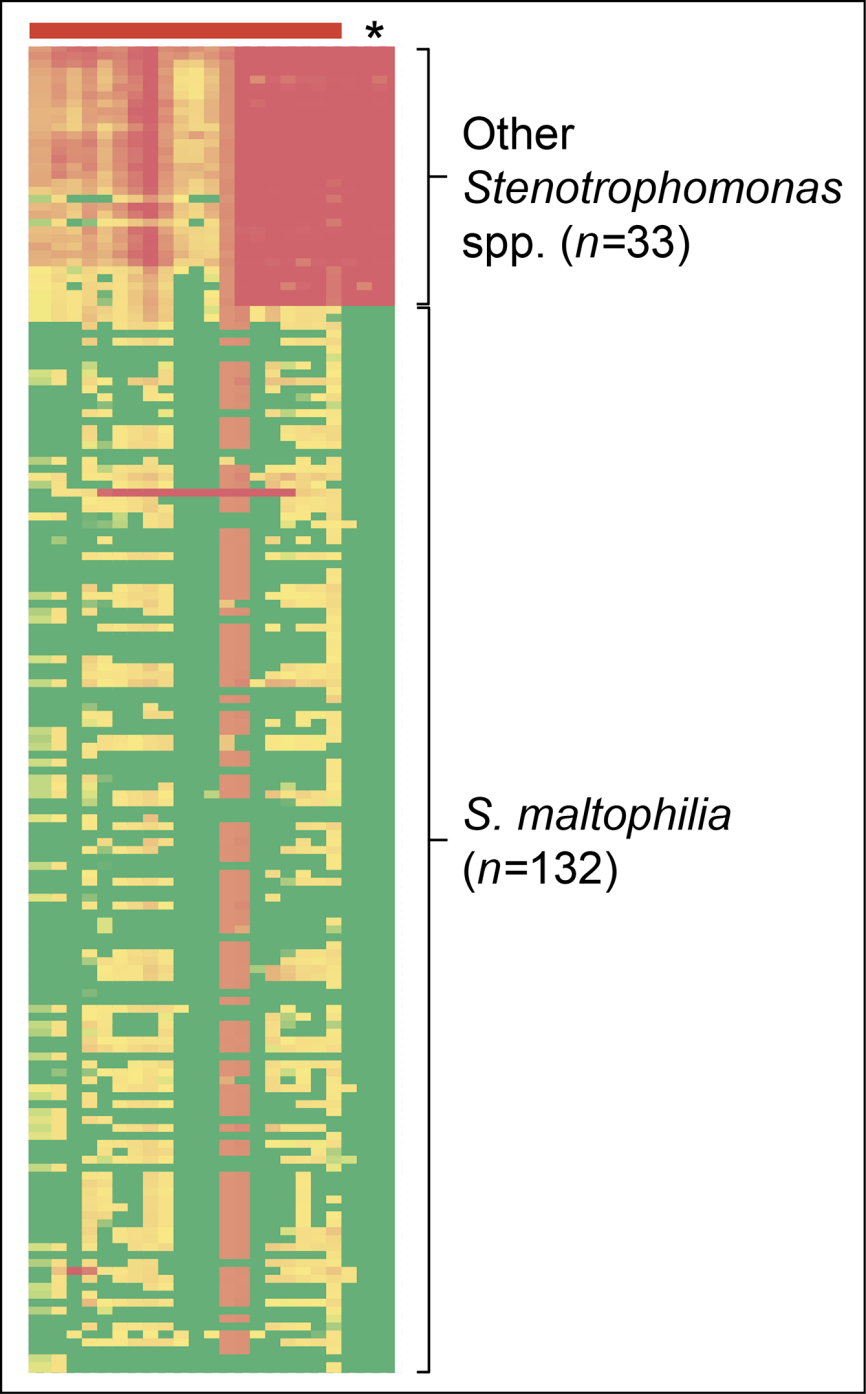
Heatmap of the SPANDx BEDcov output from *Stenotrophomonas* spp. used to identify *Stenotrophomonas maltophilia *-specific loci. A region of 6 kb in total (including a 4 kb region encoding formate dehydrogenase α, β and γ subunits) was found to be highly conserved in *S. maltophilia* but absent or highly divergent in other *Stenotrophomonas* spp., including *S. indicatrix* and *S. lactitubi* (asterisk). The 20 kb region immediately downstream of this 4 kb locus (red horizontal bar) is included for comparison. Green, >99 % coverage across the BEDcov window according to BWA-MEM read mapping; red, <20 % coverage; orange through pale green, 20–99 % coverage.

Following identification of the species boundary for *S. maltophilia *, we next identified loci specific for *S. maltophilia *. BEDcov outputs identified a conserved 4 kb region in all 132 *S *. *maltophilia* genomes that was either absent or highly divergent in the other *Stenotrophomonas* spp., including *S. indicatrix* and *S. lactitubi* (Fig. 2). The genetic coordinates for these loci are 3 935 000–3 939 000 in *S. maltophilia* K279a, spanning coding regions for formate dehydrogenase (α, β and γ subunits; encoded by *fdnG, fdnH* and *fdnI*, respectively). Formate dehydrogenase is also encoded by other pathogens, including *P. aeruginosa* [34] and *Burkholderia* spp. [35]. To account for this factor, blast analysis was next performed to identify genetic regions that had high conservation across all *S. maltophilia* strains but were divergent in all other species, including *P. aeruginosa* and *Burkholderia* spp. Using this approach, we targeted our *S. maltophilia *-specific assay to permit amplification of *S. maltophilia *, to the exclusion of all other species.

Upon identification of a suitable 344 bp *S *. *maltophilia *-specific amplicon (coordinates 3 935 993–3 936 336 in K279a; located within *fdnG*), a microbial discontiguous MegaBLAST analysis was conducted to confirm specificity (performed 15 March 2019). For all available *S. maltophilia* genomes, 100 % sequence identity for both primers and probe was attained. Unexpectedly, the genomes of two recently published *S. indicatrix* strains, which were isolated from soil in Lebanon (RS1 and RS7; GenBank accession numbers NZ_RKSQ00000000 and NZ_RKSR00000000, respectively), provided close blast hits to this amplicon, despite this locus being absent or highly divergent in the four *S. indicatrix* genomes that were used in the phylogenomic analysis. Fortunately, RS1 and RS7 contained two SNPs in the probe-binding region, including a mismatch at the very 3′ end, which would be expected to inhibit or substantially reduce fluorophore detection in these strains due to poor probe-binding kinetics.

### Origin of formate dehydrogenase loci in *S. indicatrix* RS1 and RS7

**Fig. 3.**
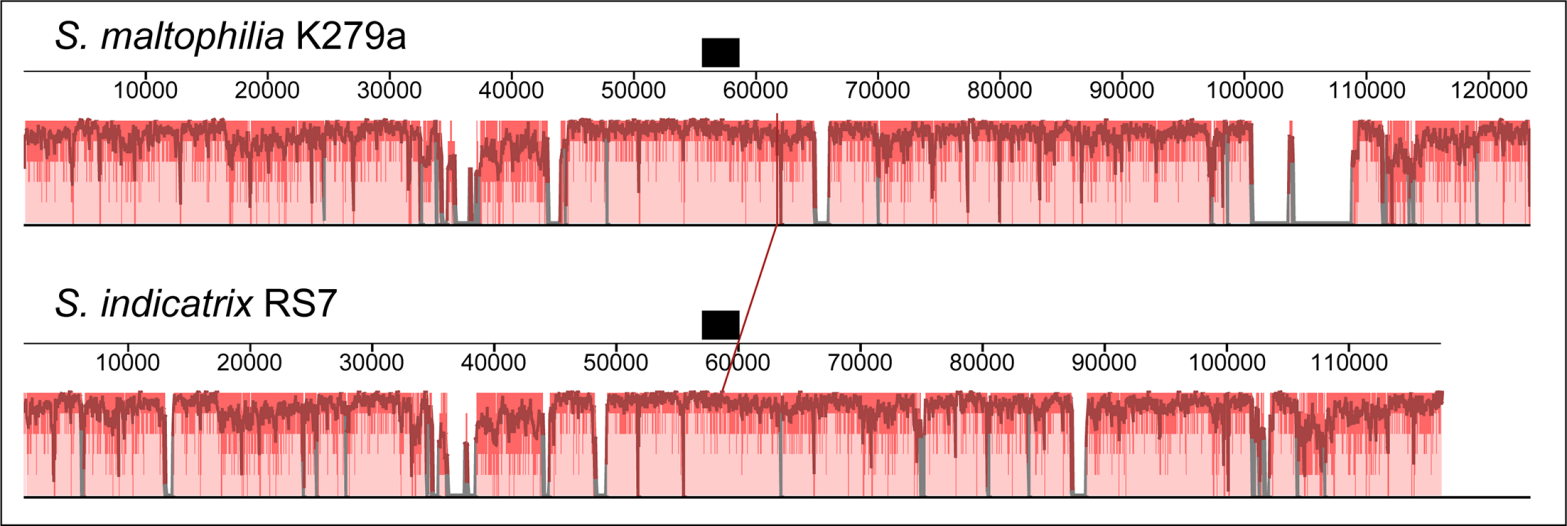
ProgressiveMauve alignment of the ~118 kb *Stenotrophomonas indicatrix* RS7 contig, RKSR01000015.1, against *Stenotrophomonas maltophilia* K279a. The region encoding the formate dehydrogenase α subunit, *fdnG*, is represented by a black bar. This analysis reveals good synteny between *S. maltophilia* and *S. indicatrix* at genetic regions both uptream and downstream of the formate dehydrogenase locus. Other *S. indicatrix* strains (not shown) lack a ~12.4 kb region compared with RS7 and *S. maltophilia* that includes this formate dehydrogenase locus.

To investigate the origin for the formate dehydrogenase loci in two of the six *S. indicatrix* strains, we examined the synteny surrounding this set of genes in *S. indicatrix* RS7 compared with *S. maltophilia* K279a. This analysis revealed that there was good synteny in both upstream and downstream flanking regions of the formate dehydrogenase loci between *S. maltophilia* and *S. indicatrix* (Fig. 3). Comparison of RS7 with *S. indicatrix* WS40 revealed that the latter strain lacked a ~12.4 kb region encompassing coordinates 53 849–66 256 in the RS7 RKSR01000015.1 contig, which includes the *fdnG* locus. Given that RS1 and RS7 are genetically highly similar (Fig. 1), and no other *S. indicatrix* possess the formate dehydrogenase loci, it is likely that a recent common ancestor of RS1 and RS7 acquired these loci from a *S. maltophilia* strain via homologous recombination, with subsequent genetic divergence occurring between *S. indicatrix* and *S. maltophilia *.

### blast analysis identifies further *S. maltophilia* misclassification

In addition to RS1 and RS7, blast analysis revealed several non-* S. maltophilia* matches to this amplicon, including three *P. geniculata* strains, several misclassified *Stenotrophomonas* spp. and even misclassifications assigned to distant bacteria e.g. *Acinetobacter baumannii* (4300STDY7045681; GenBank accession no. UFGS00000000.1), *P. aeruginosa* (E15_London_28_01_14; GenBank accession no. CVWF00000000.1) and *Pseudomonas* spp. (UBA10046; GenBank accession DPXK00000000.1). In all cases, these isolates were confirmed to be *S. maltophilia* according to Microbes blast analysis of the whole genome, thereby representing species assignment errors in the NCBI database, and demonstrating the specificity of our *S. maltophilia* target.

### *S. maltophilia* real-time PCR assay specificity

To further assess assay specificity, DNA from 89 *S *. *maltophilia* isolates collected from acute and chronic infections, including an abdominal wound (*n*=1), an abdomen aspirate (*n*=1), blood (*n*=12), a bronchial washing (*n*=1), CF sputum (*n*=26), endotracheal tubes (*n*=18), a joint swab (*n*=1), non-CF sputum (*n*=21), a portacath tip (*n*=1), an ulcer swab (*n*=1) and urine (*n*=6), were tested. The real-time PCR assay accurately detected 100 % of the *S. maltophilia* isolates but did not amplify across any of the tested non-* S. maltophilia* species [* Burkholderia thailandensis* (*n*=1), *P. aeruginosa* (*n*=11), *K. pneumoniae* (*n*=2), *K. oxytoca* (*n*=1), *E. cloacae* (*n*=4), *E. aerogenes* (*n*=1), *Staphylococcus epidermidis* (*n*=2) and *Staphylococcus aureus* (*n*=1)]. As *S. maltophilia* is known to be a common bacterium in CF sputa, we next tested the assay across 16 CF sputum samples obtained from nine patients. Of these, 10 sputa demonstrated *S. maltophilia* presence at various abundances, with 16S rRNA gene to *S. maltophilia* assay ∆*C*_t_ values ranging from 2.5 (SCHI0019 day 11; the same as pure *S. maltophilia* DNA control) to 17.0 (Table 1). In these CF sputum samples, *S. maltophilia* was frequently co-detected alongside mucoid and non-mucoid *P. aeruginosa* (Table 1). In two longitudinal samples (SCHI0020 and SCHI0021), *S. maltophilia* was only detected at very low levels, according to ∆*C*_t_ values, at the day 1 time point, with the two subsequent sputa being PCR-negative for this organism. In contrast, *S. maltophilia* persisted in the other two longitudinal samples (SCHI0002 and SCHI0019). There was an 71.4 % congruence between the two methods, with two PCR-positive sputa (SCHI0020 day 1 and SCHI0021 day 1) being negative by culture (Table 1). However, these two specimens had the lowest *S. maltophilia* load (∆*C*_t_ values of 13.8 and 17.0), indicating higher sensitivity with the real-time PCR.

**Table 1.** Real-time PCR quantification of *Stenotrophomonas maltophilia* on 16 sputa obtained from CF airways, with concurrent culture diagnosis

Sample	Δ*C*_t_*	Culture result	Other known culture growth
* S. maltophilia* isolate control	2.5	Positive	Pure isolate
SCHI0002 day 1	7.9	Positive	Mucoid and non-mucoid *P. aeruginosa *
SCHI0002 day 320	6.0	Positive	Mucoid *P. aeruginosa *
SCHI0008	na	nd	nd
SCHI0010	na	Negative	Mucoid and non-mucoid *P. aeruginosa *
SCHI0011	12.4	nd	nd
SCHI0013	9.7	nd	nd
SCHI0014	8.0	Positive	No other bacterial growth
SCHI0019 day 1	6.7	Positive	* Staphylococcus aureus *
SCHI0019 day 11	2.5	nd	nd
SCHI0019 day 46	6.5	nd	nd
SCHI0020 day 1	17.0	Negative	Mucoid *P. aeruginosa *
SCHI0020 day 6	na	nd	nd
SCHI0020 day 31	na	nd	nd
SCHI0021 day 1	13.8	Negative	MRSA, mucoid and non-mucoid *P. aeruginosa *
SCHI0021 day 5	na	nd	nd
SCHI0021 day 13	na	nd	nd

MRSA, Methicillin-resistant *Staphylococcus aureus*; na, no amplification; nd, not determined.

*Cycles-to-threshold difference.

### *S. maltophilia* real-time PCR assay sensitivity

**Fig. 4.**
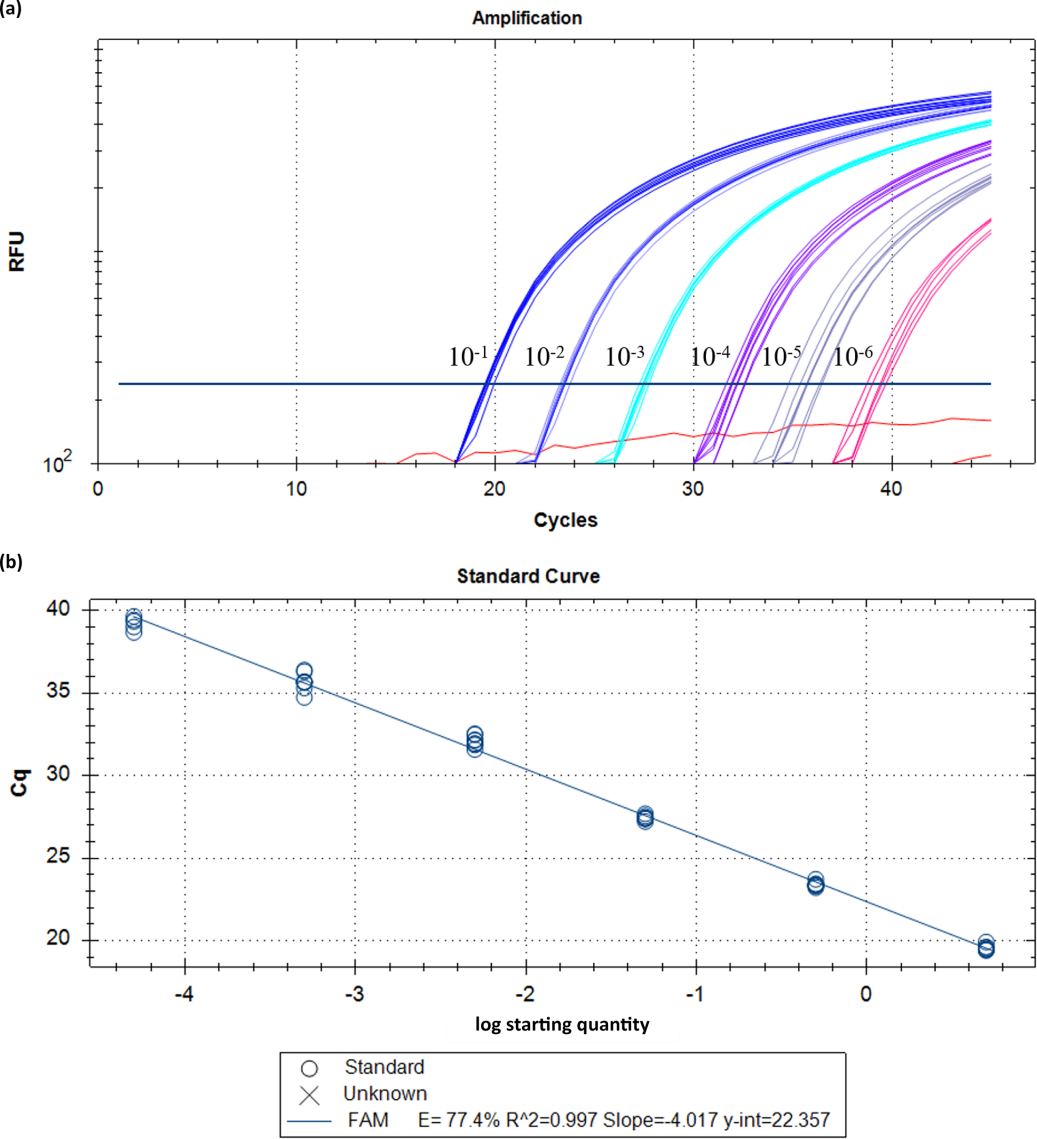
LoD and LoQ values for the *S. maltophilia-*specific real-time PCR assay. To determine the LoD and LoQ, serial dilutions of a *S. maltophilia-*positive control DNA sample were performed across eight replicates, ranging from 50 ng µl^–1^ to 0.05 fg µl^−1^ (a). To assess the correlation coefficient, a standard curve was also included for these dilutions, resulting in an *R* ^2^ of 0.997 (b). LoD and LoQ were identified as ~5 fg µl^−1^ and ~0.5 pg µl^−1^, respectively. All 24 negative controls (red) were negative. RFU, relative fluorescence units; Cq, quantification cycle (also known as *C*_t_ [cycles-to-threshold]).

To assess assay sensitivity, LoD and LoQ values were determined (Fig. 4). Using a 10-fold DNA dilution series (50 ng µl^−1^ to 0.05 fg µl^−1^), the LoD for this assay was ~5 fg µl^−1^ or ~9 genome equivalents (GEs), and the LoQ was 0.5 pg µl^−1^ or ~94 GEs.

## Discussion

*S. maltophilia* is emerging as an important multidrug-resistant nosocomial pathogen, being amongst the top three most common non-fermentative Gram-negative bacilli identified in hospitalized patients [5, 36]. Despite *S. maltophilia* being well-adapted to many environments, most infections occur in immunocompromised individuals in the nosocomial setting [10], although community-acquired infections are also on the rise [1]. Here, we describe what is to the best of our knowledge the first real-time PCR assay to detect *S. maltophilia* with 100 % accuracy in purified colonies, and demonstrate that this assay is superior to microbiological culture for detecting this multidrug-resistant bacterium in polymicrobial respiratory specimens collected from CF patients.

There is currently a lack of a rapid, cost-effective, accessible and accurate diagnostic method for *S. maltophilia* detection*,* particularly from polymicrobial clinical specimens such as CF sputa. As *S. maltophilia* is thought to be the only *Stenotrophomonas* species to cause human disease, mass spectrometry (MS)-based systems such as VITEK 2 and VITEK MS are a common diagnostic method in large, centralized pathology laboratories. However, the accuracy of species determination using MS is heavily dependent on the quality of the associated databases, and it is currently unknown whether other *Stenotrophomonas* spp. can be accurately differentiated from *S. maltophilia* on these systems. In addition, access to this instrument is limited to well-resourced laboratories owing to a large barrier-to-entry cost [~US $200 000 (£162 586)] [37–39]. Furthermore, as CF sputa are polymicrobial, overgrowth of other bacteria on selective culture plates is common. This is particularly problematic for VITEK diagnosis when *S. maltophilia* is present in low abundance or the patient is co-infected with mucoid *P. aeruginosa *. From a genotyping standpoint, 16S rRNA gene PCR has been used to identify *S. maltophilia* in blood samples for patients undergoing chemotherapy for leukemia [40], and a multiplex PCR targeting *P. aeruginosa *, *S. maltophilia* and *Burkholderia cepacia* successfully identified *S. maltophilia* in 85 % of cases [41]. However, these assays have either not been optimized to avoid non-specific amplification in other *Stenotrophomonas* spp. and members of the closely related genus *Xanthomonas *, or they require downstream processing (e.g. gel electrophoresis, Sanger sequencing) to confirm results, which is laborious, time-consuming and raises potential laboratory contamination issues.

Therefore, the purpose of this study was to use large-scale comparative genomics to identify a *S. maltophilia-*specific genetic target, and to subsequently design a highly specific and accurate real-time PCR-based assay for identifying *S. maltophilia *. Using this approach, we identified a genetic region with high specificity for *S. maltophilia *, which was subsequently targeted for assay development. We found that our newly developed assay correctly identified 89 *S *. *maltophilia* isolates with 100 % accuracy. The accuracy and specificity of this assay is both highly sensitive and selective for *S. maltophilia *, with an LoQ and LoD of ~94 and ~9 GEs, respectively. We chose the BHQ probe real-time PCR format due to its relatively inexpensive up-front cost [~US $25 000 (£30 750) for real-time instrumentation], low per-reaction cost [~US $0.80 (£0.98) per sample when performed in duplicate], high-throughput capacity, closed-tube format (which eliminates post-PCR contamination concerns), simple set-up and rapid turnaround-time (~1 h). This format also enables robust identification of target species in polymicrobial specimens. Although not examined in this study, the multi-fluorophore capacity of many real-time PCR instruments also enables multiplexing of probe-based assays for the simultaneous identification of multiple organisms in a single specimen, leading to further cost reductions.

Our *in silico* and laboratory results indicate that all non-* S. maltophilia* micro-organisms failed to amplify, with the possible exception of two *S. indicatrix* strains, RS1 and RS7, the genomes of which only became available subsequent to assay design. *S. indicatrix* is a newly identified *Stenotrophomonas* species [33] that has so far been isolated from dirty dishes in Germany (strain WS40 [33]), sewage in China (strain G4; unpublished), a rotting apple in Germany (Myb57; unpublished), soil in Lebanon (strains RS1 and RS7; unpublished) and a human respiratory infection in Germany (ICU300 [42]). Comparative analysis of RS7 and *S. maltophilia* indicated recent homologous recombination of the formate dehydrogenase locus targeted by our study; however, blastn analysis showed that there were two SNPs in the probe-binding region, including a SNP at the 5′ ultimate base of the BHQ probe, which would likely result in poor or no amplification. Taken together, we show that our assay is highly specific for *S. maltophilia *, particularly in clinical samples, but it also has applicability for testing environmental samples, such as hospital water supplies.

Although the quality of life and life expectancy for people with CF has markedly increased in recent decades due to improvements in antibiotic treatments and clinical management, persistent polymicrobial infections in CF airways remain the primary cause of morbidity and mortality [43]. A recent longitudinal study of a single CF patient’s airways using a cutting-edge metatranscriptomic approach, which measures only the ‘active’ microbial population through mRNA characterization, revealed that *S. maltophilia* was the second most prevalent bacterium behind *P. aeruginosa* in the 6 months prior to death [43]. Our results also revealed a high prevalence of *S. maltophilia* in adult CF sputa, with 10/16 samples positive for this bacterium according to our assay. As some of these sputa had concurrent culture results, we demonstrated an improvement in detection with 71.4 % congruence to real-time PCR results, with two culture-negative samples returning as positive by real-time PCR (SCHI0020 day 1 and SCHI0021 day 1). While our sample size is low, this finding demonstrates that our assay may have a higher sensitivity for detecting *S. maltophilia* in CF clinical specimens than culture methods, although it cannot be ruled out that *S. maltophilia* PCR positivity may be due to the presence of DNA in the CF sputa from a recently eradicated *S. maltophilia* infection. Of four longitudinally collected sputa, one patient (SCHI0019) had *S. maltophilia* at all time points (days 1, 11 and 46; Table 1) despite intravenous meropenem and tobramycin antibiotic therapy administered on day 2 of admission, and another patient, SCHI0002, was positive for *S. maltophilia* in samples that were collected nearly 12 months apart (days 1 and 320), indicating either long-term airway persistence or reinfection with this organism. In one sputum sample from SCHI0019 (day 11), the ∆*C*_t_ value between the 16S rRNA gene and *S. maltophilia* PCRs was identical to that of pure *S. maltophilia* culture (Table 1), indicating that *S. maltophilia* had become the dominant, and potentially sole, bacterial species in this specimen. Although outside the scope of this study, this finding demonstrates the potential for *S. maltophilia* to persist and dominate in CF airways following antibiotic-driven microbiome perturbations, which may have implications for rapid re-infection with more formidable pathogens such as *P. aeruginosa *. A further two patients, SCHI0020 and SCHI0021, had *S. maltophilia* at day 1, but subsequent samples (up to day 31 and 13, respectively) were PCR-negative following intravenous ceftazidime and tobramycin antibiotic treatment phase administered on day 2 of admission, indicating successful eradication of *S. maltophilia* in these cases. Future work will entail testing our newly developed assay across larger CF sputum panels, including longitudinal samples, to further examine the potential mutualistic relationships between *S. maltophilia* and other pathogens such as *P. aeruginosa *, and to assess assay performance directly on clinical specimens to further reduce sample processing timeframes.

In conclusion, the ability to accurately, rapidly and cheaply detect *S. maltophilia* is critical for understanding the prevalence of this underappreciated opportunistic pathogen and for reducing its burden of disease. The implementation of this assay in the clinical setting will enable researchers, clinicians and pathologists to more accurately identify this multidrug-resistant bacterium, particularly in isolates that have been ruled out as other multidrug-resistant Gram-negative pathogens, such as *P. aeruginosa* or *Burkholderia* spp. Finally, the correct and rapid identification of *S. maltophilia* will improve antibiotic stewardship measures by enabling more targeted eradication of this pathogen, and in polymicrobial infections such as those commonly found in CF airways, *S. maltophilia* eradication may reduce the prevalence and persistence of more serious pathogens such as *P. aeruginosa *, leading to improved quality of life and lifespans for people with CF.

## Data bibliography

1. Hagstrom A *et al*. BioProject PRJNA19369 (2008).

2. Lira F *et al*. BioProject PRJEA89665 (2012).

3. Lucas S *et al*. BioProject PRJNA17107 (2013).

4. DOE Joint Genome Institute. BioProject PRJNA185300 (2013).

5. Lucas S *et al*. BioProject PRJNA53943 (2013).

6. Roach D.J. *et al*. BioProject PRJNA267549 (2014).

7. Kanamori H *et al*. BioProject RJDB3841 (2015).

8. Sanger BioProject PRJEB6891 (2015).

9. Tang H *et al*. BioProject PRJNA156719 (2015).

10. Pak TR *et al*. BioProject PRJNA277366 (2015).

11. Saffarian A *et al*. BioProject PRJNA278502 (2015).

12. Patil PP *et al*. BioProject PRJNA284363, PRJNA284364, PRJNA284366(2015).

13. Ormerod K. BioProject PRJNA285410 (2015).

14. Vinuesa P *et al*. BioProject PRJNA296415 (2015).

15. Crossman LC *et al*. BioProject PRJNA30351 (2015).

16. Liu W. BioProject PRJEB15263 (2016).

17. Varghese N. BioProjectPRJEB16042, PRJEB17324 (2016).

18. Mitreva M *et al*. BioProject PRJNA269850, PRJNA269851, PRJNA272632 (2016).

19. Patil PP *et al*. BioProject PRJNA284369, PRJNA284375, PRJNA284376, PRJNA284378, PRJNA299446 (2016).

20. Pan X *et al*. BioProject PRJNA286061 (2016).

21. Park H. BioProject PRJNA310387 (2016).

22. Nazaret S, Bodilis J. BioProject PRJNA323844 (2016).

23. Wei Y *et al*. BioProject PRJNA326321 (2016).

24. Blow F, Darby AC. BioProject PRJNA326914 (2016).

25. Oh D-K, Kim KR. BioProject PRJNA330867 (2016).

26. Kozyreva V. BioProject PRJNA341407 (2016).

27. Richard D. BioProject RJNA344031 (2016).

28. Hattie C. BioProject PRJNA344912 (2016).

29. Niu B *et al*. BioProject PRJNA357031 (2016).

30. Varghese N. BioProject PRJEB21383, PRJEB22431 (2017).

31. Sanger BioProject PRJEB8666 (2017).

32. Patil PP *et al*. BioProject PRJNA299448 (2017).

33. Dong H *et al*. BioProject PRJNA321363 (2017).

34. Ge S. BioProject PRJNA347873 (2017).

35. Parks DH *et al*. BioProject PRJNA348753 (2017).

36. Esposito A et al. BioProject PRJNA350620 (2017).

37. Mohapatra B, Sar P. BioProject PRJNA352524 (2017).

38. Aslam F, Yasmin A. BioProject PRJNA358642 (2017).

39. Zhineng W. BioProject PRJNA388045 (2017).

40. Fouts D *et al*. BioProject PRJNA390523 (2017).

41. Weber M et al. BioProject PRJNA414363 (2017).

42. Castro-Jaimes S *et al*. BioProject PRJNA421960 (2017).

43. Sanger BioProject PRJEB20809 (2018).

44. Sichtig H *et al*. BioProject PRJEB20827, PRJNA231221 (2018).

45. Riera N *et al*. BioProject PRJNA369256 (2018).

46. Ho BC. BioProject PRJNA374779 (2018).

47. Glady-Croue J *et al*. BioProject PRJNA381518 (2018).

48. Yang W. BioProject PRJNA400855 (2018).

49. Garrido-Sanz D *et al*. BioProject PRJNA412168 (2018).

50. Parks DH *et al*. BioProject PRJNA417962 (2018).

51. Medina-Cordoba LK *et al*. BioProject PRJNA418312 (2018).

52. Chen M-X. BioProject PRJNA427609 (2018).

53. Kenzaka T, Tani K. BioProject PRJNA430144 (2018).

54. Xiong W. BioProject PRJNA437214 (2018).

55. Pelletier D *et al*. BioProject PRJNA440801 (2018).

56. Xu J. BioProject PRJNA445756 (2018).

57. Venturi V *et al*. BioProject PRJNA463055 (2018).

58. Xiong W. BioProject PRJNA474584 (2018).

59. Cao G. BioProject PRJNA486733 (2018).

60. Cooper A. BioProject PRJNA489399 (2018).

61. Li R. BioProject PRJNA505368 (2018).

62. Fouts DE *et al*. BioProject PRJNA508495 (2018).

63. Tokajian ST, Khnayzer RS. BioProject PRJNA496409, PRJNA496522 (2019).

## Supplementary Data

Supplementary File 1
